# Valorisation of baryte tailings for radiation shielding in plastics and nuclear waste disposal

**DOI:** 10.1016/j.heliyon.2024.e25719

**Published:** 2024-02-02

**Authors:** Päivi Kinnunen, Jani Pelto, Pekka Viitanen, Markus Olin, Matti Nieminen

**Affiliations:** a VTT Technical Research Centre of Finland Ltd, Visiokatu 4, 33101, Tampere, Finland; b VTT Technical Research Centre of Finland Ltd, Kivimiehentie 3, 02150, Espoo, Finland; c VTT Technical Research Centre of Finland Ltd, Tietotie 4C, 02150, Espoo, Finland

**Keywords:** Waste valorisation, Baryte, Tailings, Circular economy, Secondary material, Radiation shielding

## Abstract

Baryte (BaSO_4_) is a critical raw material with no functional recycling since the used applications are dissipative. Significant quantities of baryte end up in tailings as a side stream from the mining industry. Baryte from a secondary raw material source was used as a filler in plastics for low duty radiation shielding and as an aggregate in radiation shielding geopolymers needed for safely storing low-radioactive waste ash. Mechanical strength in geopolymers remained at a high level with 0–50 % baryte additions. Low-cost plastic composites with baryte additions showed promising attenuation for X-rays and gamma-rays. The results showed improved qualities in the direct use of the secondary baryte material in concrete and plastics in comparison to primary baryte. Baryte from an industrial waste stream was shown to be applicable to be used in radiation shielding in geopolymers for storing low-radioactive waste, and in plastics. Primary baryte can be replaced with secondary baryte to bring environmental, economic, and even functional benefits.

## 1 Introduction

Baryte is a naturally occurring mineral (BaSO_4_), which is mined [1] to be used in drilling mud, medical industry, glass making, paint and rubber industry, industrial chemicals, and in other applications, such as plastics [2]. Due to its importance to industry and restricted supplies to the market, baryte belongs to the critical raw material list in the European Union [3].

**Fig. 1:**
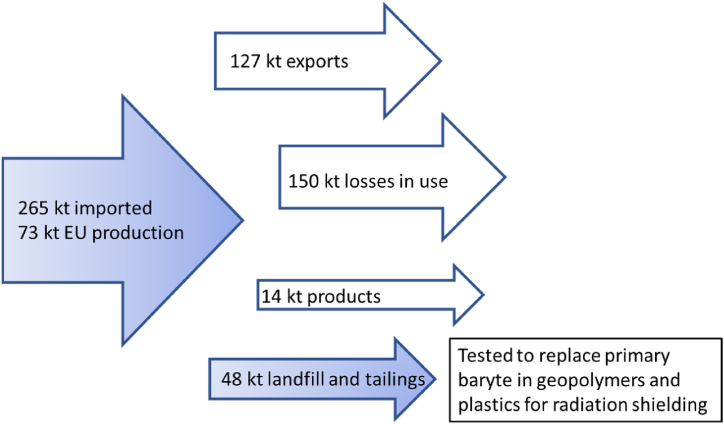
Baryte imports, use and exports in the EU (without the UK) in 2016 showing landfill and tailings as an alternative secondary source of baryte (data from Ref. [1]). This study focused on valorisation of baryte stream deposited in landfills and tailings.

No secondary sources of baryte have been identified so far, as the applications are dissipative, and no recycling takes place [1]. In addition to being mined on purpose, baryte is a common gangue mineral in other deposits, such as lead, fluorite, zinc, gold and rare earth minerals [2]. Based on material flow evaluations, a significant part of baryte ends up in landfills and tailings providing an alternative and still unused secondary source of baryte (Fig. 1). Various circular economy strategies have been proposed to valorise tailings to recover not only the metal content, but also the value of remaining minerals in higher value products [4,5]. Baryte containing tailings have attracted companies’ interests for baryte recovery. Baryte concentrate has been successfully produced from tailings in the laboratory experiments [6,7]. Valorisation of tailings would also bring added value based on lower costs of rehabilitation of old mines. However, the processing costs to remove the existing impurities of waste baryte have previously hindered its further use in conventional applications. If baryte tailings could be used without extensive refining in novel applications, such as radiation shielding, it would reduce the costs and add benefits.

Some of biologically dangerous radiation types are stopped by paper (alpha-rays) and by a layer of aluminum (beta-rays), but the propagation of X-rays and gamma-rays as high-energy electromagnetic radiation of very high frequencies is proportional to the atomic mass of the shielding material [8]. Attenuation in radiation shielding is roughly proportional to the electron and mass densities of the shielding material due to the differential interaction mechanisms of X-rays and gamma-rays with matter. The higher the concentration of heavy absorbing atoms, the more attenuation per areal mass (mass density) can be achieved, that is a higher so-called mass attenuation coefficient for a specific photon energy of the radiation [9]. The mass attenuation coefficient has a simple relation (μ/mass density) to the linear attenuation coefficient (μ) of Lambert-Beer law (Ln(I_0_/I) = -μx, where I_0_/I are measured intensities of radiation beam and x is absorbing material's thickness). Both the linear attenuation coefficient and the mass attenuation coefficient are widely applied in describing the attenuation of ionizing radiation in a narrow beam experimental setup similar to this study [10,11]. Chemical elements such as barium, bismuth, tungsten and lead with high mass density, high atomic number (electron density) and high binding energy for electrons are good absorbers of ionizing radiation.

Radiation shielding materials have versatile applications in nuclear medicine, medical imaging, nuclear power plants and nuclear waste disposal. High density concrete provides resistance towards radiation (having high linear attenuation coefficient) and is used in nuclear powerplants and reactors [8,12]. The reduction in the gamma transmission rate is significantly dependent on the density of concrete [13]. The density of normal-weight concrete is approximately 2400 kg/m^3^ and of high-density concrete >2900 kg/m^3^, which can be obtained using heavyweight aggregates [8]. The density of heavy aggregates in high density concrete needs to be over 3000 kg/m^3^ and natural minerals including magnetite, hematite, limonite and baryte belong to this group. Industrial wastes, such as slags and mining wastes, have been tested for a partial or complete replacement of conventional constituents in radiation shielding concrete [14]. When baryte was added as additive to mortar with an increase in the additive ratio, the compressive and bending strengths decreased, whereas the linear attenuation coefficient increased, and the material was effective to prevent radiation transmission [15]. Natural fine aggregates have been partially replaced up to 30 % by baryte-fluorspar tailings, and mortar with 25 % tailings had a higher compressive strength compared to the ordinary mortar [16]. The maximum compressive strength using baryte and fluorspar mine waste in the cement mortar was obtained with a 25 % replacement also in the study of Tyagi et al. [17].

Waste material comprising of low and/or medium level radioactive waste is usually encapsulated into a matrix, often Portland cement, inside a steel container prior to storage or disposal in specially planned repositories for nuclear waste. A major part of the encapsulation comprises of the matrix [18]. Typically, only about 10 wt-% of the total mass of the encapsulation is radioactive waste material, i.e., the loading factor is only approximately 10 %. However, considering requirements for long-term safety, it is possible to replace cement with a geopolymer composing partly of baryte. Geopolymers can have both higher loading factors and better waste matrix properties for long term safety than cement or bitumen-based materials. Baryte additions on the other hand can decrease radiation doses during the operation of waste containers. Adding baryte as a substance to increase radiation shielding into the encapsulating matrix can decrease the total volume of radioactive waste to be deposited and thus the associated costs.

A whole different application field for radiation shielding takes place in hospitals, X-ray and other medical imaging devices and protective casings in equipment manufactured by injection molding or extrusion-thermoforming of filled plastics. When used in low concentrations as a radiation absorbing filler in acrylonitrile butadiene styrene (ABS) thermoplastic composites, baryte at the most optimal filling ratio of 10 % has been shown to enhance tensile strength and hardness, decrease impact energy, and cause no clear change for melt-flow index [19].

This research studies the use of baryte obtained from a secondary raw material source as a filler in plastics for low duty radiation shielding and as aggregate in radiation shielding geopolymers needed for safely storing low-radioactive waste ash. The research was limited to mechanical strength and radiation shielding properties of the produced geopolymers and plastics. In the future studies, other properties e.g. leachability should be evaluated. Absorption of X-ray (Am-241, 59.5 keV; Co-57, 122.1 keV and 136.5 keV) and gamma-ray (Cs-137, 661.7 keV) photons in composites with a high loading (70–95 wt-%) of baryte enrichment in organic and inorganic binders was investigated. The purity of baryte in these applications can be modest and contain some other residual elements, which might still be present in the tailings. This can be in fact an advantage as the residual silicates in the tailings facilitate binding of the otherwise inert BaSO_4_ in inorganic or organic matrices. In the best case, the impurities of the baryte waste stream bring positive properties and added value to the targeted radiation shielding applications compared to virgin materials. This research supports the circular economy approach, where the currently unused tailings residues can be upcycled to a higher value product to add functionalities to the materials and at the same time to reduce the environmental impacts.

## 2 Material and methods

### 2.1 Characteristics of baryte sample

Mine tailings with concentration of 70–80 % or 94–95 % (by XRF) of barium sulfate were used in the experiments. The remaining part of the less concentrated sample was silicates, and the sample could be sintered at 1100–1400 °C temperatures into porous pellets having a density between 2.9 and 3.1 g/cm^3^ (theoretical density of BaSO_4_ is about 4.49 g/cm^3^). The more concentrated >94 % baryte sample was investigated as a filler with inorganic and polymer binders.

### 2.2 Baryte as a filler in binder matrices

>94 % baryte sample was used as a filler in various binder matrices (experiments A–D). In the experiment A) 20 % potassium aluminate cement (CA14) was mixed with baryte in excess water, forming a slurry. After hardening of the cement in mold the final density of the dry pellets was 2.8 g/cm^3^. In the experiment B) weighted amount of polymer polyvinyl butyral (PVB) was dissolved in 45 mL of water-ethanol (85/15 w-%) and mechanically mixed with 95 g of baryte into a homogeneous slurry. The final concentrations of PVB binder in two studied compositions were 5 and 7 wt-%. The slurry was cast into a silicone mold to form disks of approximately 1–1.5 cm in thickness. After evaporation of the solvent, the dry cake was placed in an oven at 140 °C for 1 h. The final densities of the pellets with 5 % and 7 % PVB were 2.3 g/cm^3^ and 2.5 g/cm^3^, respectively. In the experiment C) 30 wt-% alkaline borax glass frit (Varnia, Finland) was mixed with baryte and thermally sintered. In the experiment D) 25 % thermoplastic high-density polyethylene-polypropylene (HDPE-PP) was melt compounded with baryte in DSM Xplore micro-compounding extruder at 200 °C for 3–4 min. Test sample slabs of 3 mm in thickness were injection molded using a pneumatic ThermoHaake Minijet injection molding machine.

### 2.3 Baryte as an aggregate in geopolymers

Geopolymer test specimens containing ash from thermal treatment of a cesium (stable isotope) doped ion exchange resin [18] were produced adding different percentages of baryte into the geopolymer matrix (Table 1). Five samples (40x40 × 160 mm) including different amounts of baryte (5–75 wt-%) were cast in addition to one blank sample and five reference samples including different percentages of quartz sand. Mechanical strength was measured from produced specimens following CEN EN 1015–11:2019 (Methods of test for mortar for masonry - Part 11: Determination of flexural and compressive strength of hardened mortar procedure) to determine maximum possible percentage of baryte in geopolymer without losing excellent strength.

**Table 1:** Casted samples with various amounts of baryte (0–75 %) as aggregate in geopolymer.

Sample	Metakaolin (kg/m^3^)	Ash (kg/m^3^)	NaSiO_3_ (kg/m^3^)	KOH (kg/m^3^)	Baryte (kg/m^3^)
0	755	23	906	151	0
5 %	717	22	861	143	220
10 %	680	20	815	136	440
25 %	566	17	680	113	1100
50 %	378	11	453	76	2200
75 %	189	6	227	38	3300

### 2.4 Measurement of attenuation properties of baryte filler material

When baryte was used as a filler in binder matrices, radiation shielding of the specimens was tested experimentally. Measurements were done with unique samples prepared in laboratory using a small amount of available experimental baryte rich sample powders. Radiation point sources were Am-241 (59.5 keV), Co-57 (122.1 keV and 136.5 keV) and Cs-137 (661.7 keV). In the experiments with HDPE-baryte, Ba-131 was used instead. Absorption of Ba-131 X-ray and gamma-ray peaks at 30.6 keV, 31 keV, 35 keV, 35.9 keV, 79.6 keV, 81 keV, 276.4 keV, 302.9 keV, 356 keV and 383.9 keV were investigated. Point radiation sources having activities of approximately 370–400 kBq were placed in front of a collimator. The collimated beams were then passed through the samples into a gamma-spectrometer (Canberran ISOCS) equipped with a broad energy (3 keV- 3 MeV) germanium detector (HPGe BE2020 crystal) located at 0.23 m distance from the radiation source. According to the specifications, this detector provides 0.65 keV resolution at 122 keV. Measurements were done once (N = 1) for each unique sample having a definite mass density and sample thickness, or for the stacked samples of the same type of material. The largest uncertainties of the experimental setup were the effective sample uniformity and sample thicknesses, which combined had an estimated uncertainty of 5 %.

Signal integration time varied between approximately 15 min and 20 h, depending on the sample characteristics and the properties of the radiation sources. Collection time was adjusted to collect well above 10000 counts at the detector in all cases. To eliminate uncertainties from the measurement, the natural background signal was collected over 50 h. In all cases, the background signal counts were estimated 3–5 orders of magnitude lower than the signal measured in the presence of the radiation source. Assuming the Poisson statistics, the uncertainty in photon counting and the calculated reduction of beam intensity over sufficiently long measuring time (steady state) is simply a square root of a number of counts at the detector. The total experimental error of the linear attenuation and mass attenuation coefficients can be estimated from the total differentials of their expressions.

In addition, various radiation shielding parameters were calculated. The half-value layer (HVL) representing the thickness reducing a particular radiation by one-half of the level of intensity was calculated as *HVL* = ln(2)/μ [20], and the tenth-value layer (TVL) *TVL* = ln(10)/μ. The linear and mass attenuation coefficients were calculated from the photon counts of attenuated gamma-ray peaks according to the Lambert-Beer law; linear attenuation coefficient μ = Ln (I0/I)/x and mass attenuation coefficient (μ/mass density).

### 2.5 Dose rates of waste drums with different filling materials

Modelling was done to simulate actual packages for low and intermediate nuclear waste. The base case has high, but still plausible amounts of Co-60 and Cs-137 in a waste drum [21]. Cases 0–2 present incinerated waste with 10-fold activities. Radiation levels were calculated for a container (height 80 cm, diameter 56.6 cm, material 1 mm iron) with the distance of 10 cm ja 1 m from the surface of the container. Modelling was done using the MicroShield™ program, which is widely used for radiation shielding calculations using the Point-Kernel method. Homogenous density and activity distribution were assumed in calculations. The 10 cm value represents the radiation dose rate at the surface of the package and the dose rate at 1 m distance is important when considering operational doses and transport of the packages.

## 3 Results and discussion

### 3.1 Sintered baryte and baryte composites

The studied thermally sintered and cast samples were slightly porous and hence their densities were lower than theoretically predicted. The densities of sintered baryte composites are given in Table 2. Theoretical density of BaSO_4_ is 4.49 g/cm^3^. Secondary baryte from tailings contained a high amount of residual silicate impurities which facilitated thermal sintering. Silicates have been shown to function as fillers in polymer composites preferentially when uniformly dispersed, and a chemical bond between the polymer and silica filler can be formed [22,23]. This research focused on radiation shielding applications. It is noteworthy that the successful use of baryte as a functional filler in various composites can be beneficial also in other applications. For example, plastic composites are used in noise protection as soundproofing material. In soundproofing, the mass law states that the highest sound insulation is obtained with materials of highest densities as the high mass makes the vibration of the material more challenging [24]. Therefore, the high-density composites obtained in this study could be investigated for sound insulation purposes in the future research.

**Table 2:** Mass densities of studied composite materials. HDPE= high-density polyethylene-polypropylene; CEM=cement; PVB= polymer polyvinyl butyral.

Binder material/loading %	Baryte in composite (wt-%)	Density (g/cm^3^)
–	100 (thermally sintered)	3.0
HDPE 25 %	75	2.25
CEM 10 %	90	2.82
CEM 20 %	80	2.77
glass frit 30 %	70	2.64
PVB 5 %	95	2.51
PVB 7 %	93	2.32
reference Lead (100 %) sheet	0	11.34

### 3.2 Baryte geopolymers

**Fig. 2:**
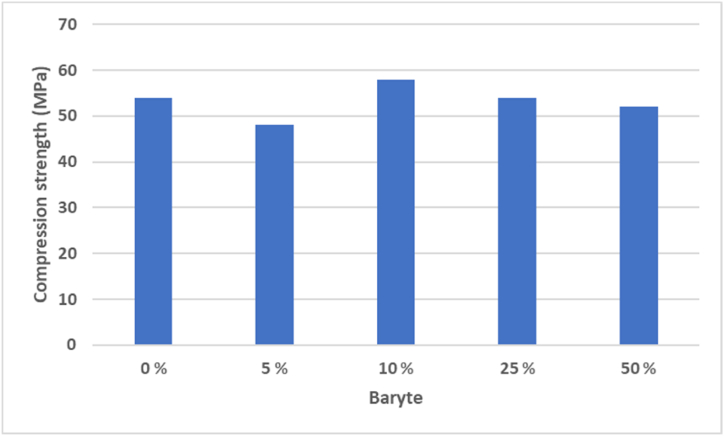
The effect of baryte addition (0–50 %) on the mechanical strength of geopolymer specimens.

The preparation of geopolymer samples was possible with 0–50 wt-% baryte additions. When continuing adding 75 wt-% baryte, there was an upper limit to the baryte content, and the preparation of the specimen was not possible. Fig. 2 shows the compression strength of cast specimens after 7 days. The results show that baryte acts as an inert aggregate and the additions of baryte below 50 wt-% do not significantly affect the mechanical strength of the produced geopolymers. In radioactive structures, mechanical strength [8] and heterogeneity of the material composition [9] are important parameters in addition to density. The compressive strength has both decreased [15] and increased [16] with baryte additions to mortars. Using baryte as an aggregate (0–100 %) did not have a direct impact on mechanical durability of concrete [25].

### 3.3 Attenuation of X-rays and gamma-rays in sintered baryte and in baryte composites

**Fig. 3:**
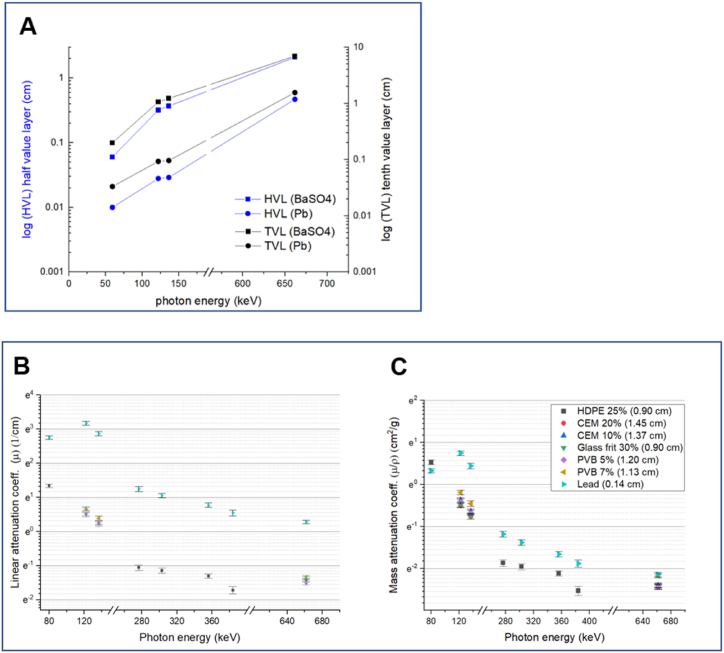
A) Half-value layer (HVL) and tenth-value layer (TVL) of sintered baryte disk (density 3.0 g/cm^3^ and approx. 75 % baryte concentration) sample and lead as reference; B) linear attenuation coefficient, and C) mass attenuation coefficient of the sample composite materials. Reference: Lead sheet of thickness 1.4 mm, density 11.34 g/cm^3^.

Attenuation of X-rays and gamma-rays in sintered baryte disks were quantified versus lead (Fig. 3A). Attenuation of four photon wavelengths in the baryte composites is shown in Fig. 3B and C. As expected, the results show that X-ray and gamma-ray attenuation for baryte is lower than for lead for the range of studied photon energies. The HVL values increase with increased energy. With the photon energy of 650 keV, the baryte material thickness needs to be 4.5 times higher than with pure lead. For lower photon energies, the half and tenth value thicknesses are smaller. Linear attenuation coefficients and mass attenuation coefficients of baryte containing polymer specimens were lower compared to lead, but still reasonably high to provide high enough radiation shielding to various applications. In the case of typical low and intermediate nuclear waste the dimensioning must be done for the higher energies. Thus, shielding thicknesses will be quite adequate when considering lower energies.

### 3.4 Attenuation of radiation in baryte geopolymers

Radiation levels calculated for a container (height 80 cm, diameter 56.6 cm, material 1 mm iron) with the distance of 10 cm and 1 m from the surface are shown in Table 3. The reduction in radiation level is approximately 36–37 %, when geopolymerization and baryte addition (70 %) are used. When more baryte is added to the mass, the reduction of radiation level would be higher, even more than 50 %. Thermal treatment of radioactive waste can be used for efficient decrease of mass up to 100 vol reduction factor and for protecting from unwanted leaching [26]. The calculation showed that baryte geopolymer could be used for radioactive shielding of thermal ash in waste handling. For real applications it must be noted that the radiation levels are high and require the usage of remote handling. Another practical aspect of heavy filling is that the weight of drums may become too heavy for the packaging and handling equipment.

**Table 3:** Radiation levels calculated for a waste container with concrete, geopolymerization and baryte addition of 70 w-% at standard and 10 times higher activity levels. Case 1 (1000 g metakaolin; 126.6 g gasified resin; 1177 g sodium silicate; 202 g potassium hydroxide), Case 2 (1000 g metakaolin; 30 g gasified resin; 1200 g sodium silicate; 202 g potassium hydroxide; 5827 g baryte). The activities are arbitrarily chosen to demonstrate radiation attenuation and are not compatible with the amount of gasified resin in the samples.

Radiation level (mSv/h)			Reduction in radiation level (%) using case 2	
	10 cm	100 cm	10 cm	100 cm
*Base case:* Activity 0,3 + 0,3 TBq Concrete casting	296	28		
*Case 0:* 10 x activity, concrete casting	2821	272	36.1	35.6
*Case 1:* 10 x activity, Geopolymerization + concrete casting	2864	276	37.1	36.6
*Case 2:* 10 x activity, Geopolymerization + baryte	1802	175		

Other aspects for using the generated composites as binder material for radioactive ash were also promising. Typical waste acceptance criteria are mechanical strength and low leachability as well as the resistance against thermal impacts [27,28]. The above noted chemical inertness and high-level mechanical strength encourage further testing of the generated waste products. One of the foreseen tests is to check the stability of the material after irradiation with radiation doses which would accumulate in the final disposal.

## 4 Conclusions

The results show the possibility to valorise baryte from tailings in high value applications and to replace primary baryte with circular one. Enriched baryte from tailings contained a high amount of residual silicate impurities which facilitated thermal sintering of baryte. Since high purity baryte is chemically rather inert, the silicate impurities also facilitate good adhesion to many kinds of organic and inorganic binder matrices. The generated low-cost plastic composites in the density range of 2.6–2.9 g/cm^3^ showed promising attenuation for X-rays (30–100 keV) and gamma-rays (100–660 keV), which can be applied in large volume applications, such as radiation shielding in building walls in the storage and handling of nuclear waste and in nuclear medicine facilities. Using secondary baryte in geopolymers in nuclear waste ash disposal was also shown as a potential solution. The mechanical strength in geopolymers remained at a high level, when 0–50 % baryte was added.

The radiation calculations showed that the radiation levels and volumes of the waste can be significantly lowered, which brings economic benefits to the low-level nuclear waste predisposal operations.

All results showed that the selected applications were suitable for baryte side stream utilization and there was no need to remove the impurities. The possibility to use side streams, which still contain some impurities in the matrix, enhances the economics since the costly concentration and purification processes can be omitted. In fact, silicate impurities added benefits in these applications. Typically, mineral residues from mining are used in low value applications. This study showed that also high value applications are possible. The valorisation of baryte tailings in radiation shielding applications has the potential to decrease the use of primary baryte from mining, and to bring environmental, economic, and technological benefits.

## Data availability statement

Data associated with the study has not been deposited into a publicly available repository. Data has been included in this article.

## Ethics statement

Review and/or approval by an ethics committee was not needed for this study because the study did not contain human research.

## CRediT authorship contribution statement

**Päivi Kinnunen:** Conceptualization, Funding acquisition, Investigation, Project administration, Writing – original draft, Writing – review & editing. **Jani Pelto:** Conceptualization, Investigation, Methodology, Visualization, Writing – original draft, Writing – review & editing. **Pekka Viitanen:** Investigation, Methodology, Writing – original draft, Writing – review & editing. **Markus Olin:** Supervision. **Matti Nieminen:** Conceptualization, Funding acquisition, Methodology, Supervision.

## Declaration of competing interest

The authors declare that they have no known competing financial interests or personal relationships that could have appeared to influence the work reported in this paper.
